# Cataract surgery and dry eye disease: A review

**DOI:** 10.1177/1120672120929958

**Published:** 2020-06-09

**Authors:** Khayam Naderi, Jack Gormley, David O’Brart

**Affiliations:** 1 Department of Ophthalmology, Guy’s and St Thomas’ NHS Foundation Trust, London, UK; 2 King’s College London, London, UK

**Keywords:** Cataract surgery, dry eye disease

## Abstract

**Aim:**

To review published literature concerning cataract surgery and dry eye disease (DED).

**Methods:**

A search was undertaken using the following: PubMed (all years), Web of Science (all years), Ovid MEDLINE(R) (1946 to 12 December 2019), Ovid MEDLINE(R) Daily Update 10 December 2019, MEDLINE and MEDLINE non-indexed items, Embase (1974–2019, week 49), Ovid MEDLINE (R) and Epub Ahead of Print, In-Process and Other Non-Indexed Citations and Daily (1946 to 12 December 2019), CENTRAL (including Cochrane Eyes and Vision Trials Register; Cochrane Library: Issue 12 of 12 December 2019), metaRegister of Controlled Trials (mRCT) (www.controlled-trials.com), ClinicalTrials.gov (www.clinicaltrial.gov) and WHO International Clinical Trials Registry Platform (www.who.int/ictrp/search/en). Search terms included ‘cataract surgery’, ‘phacoemulsification’ and ‘cataract extraction’, combined with ‘dry eyes’ and ‘ocular surface’. Relevant in-article references not returned in our searches were also considered.

**Results:**

Publications identified included systematic reviews, meta-analysis, randomized controlled trials, cohort studies, case series and laboratory-based studies. Published data highlighting the burden of DED both prior and following cataract surgery were reviewed as well as studies highlighting the effects of cataract surgery on the ocular surface, intra-operative measures to reduce deleterious effects on the ocular surface and current evidence on the management options of post-operative DED.

**Conclusions:**

DED is common and can be exacerbated by cataract surgery. Ophthalmologists need to assess for pre-existing DED and instigate treatment before surgery; be aware of reduced accuracy of measurements for surgical planning in the presence of DED; limit intra-operative surgical factors damaging to the ocular surface; and consider management to reduce DED post-operatively.

## Introduction

The development of visually symptomatic cataract is common. Cataract surgery is one of the most frequent and successful interventions currently undertaken in medicine with approximately 434,000 cataract operations performed annually in England and Wales.^1,^^2^ Modern small-incision cataract surgery offers excellent clinical outcomes coupled with rapid post-operative recovery and low risk of complications.^3^ As such, it is accompanied by ever-increasing surgeon and patient expectations.^4,^^5^ Although the technological breakthroughs in cataract surgery over the past half century have had a positive impact on the quality of life (QOL) of millions of individuals around the world, there are potential complications, which in cataract surgery may be both sight and non-sight threatening.^3^

While research, clinical and technological developments tend to be focused on the prevention of sight-threatening complications, it is important that they do not neglect the avoidance and minimization of non-sight-threatening adverse events, as these can significantly impact on patient QOL. Such an example is dry eye disease (DED), such that the detrimental effects of cataract surgery on the ocular surface can both directly cause and exacerbate pre-existing DED.^6,^^7^ This is important not only with reference to symptomatology and complications of DED itself, such as increased risk of infections, but also with regards to the accuracy of pre-operative assessments. Precise topography, tonometry and biometric measurements are prerequisites for surgical planning^8,^^9^ and eventual post-operative visual performance. They require, as the first refractive component of the eye, an intact, healthy pre-corneal tear film.^10^

The aim of this narrative review is to assess the intra-operative factors in cataract surgery which affect the ocular surface, particularly in relation to the development and exacerbation of pre-existing DED. The evidence surrounding pre-operative and intra-operative considerations to limit harmful effects on the ocular surface will be reviewed, as will potential post-operative management options in the treatment of DED following cataract surgery.

## Methods

A search was undertaken using the following databases: PubMed (all years), the Web of Science (all years), Ovid MEDLINE (R) (1946 to 12 December, 2019), Ovid MEDLINE (R) Daily Update 10 December 2019, MEDLINE and MEDLINE non-indexed items, Embase (1974–2019, week 49), Ovid MEDLINE (R) and Epub Ahead of Print, in-Process & Other Non-Indexed Citations and Daily (1946 to 12 December 2019), CENTRAL (including Cochrane Eyes and Vision Trials Register; Cochrane Library: Issue 12 of 12 December 2019), metaRegister of Controlled Trials (mRCT) (www.controlled-trials.com), ClinicalTrials.gov (www.clinicaltrial.gov) and WHO International Clinical Trials Registry Platform (www.who.int/ictrp/search/en). Search terms included ‘cataract surgery’, ‘phacoemulsification’ and ‘cataract extraction’, combined with ‘dry eyes’ and ‘ocular surface’. Published articles in English were preferentially selected. Relevant in-article references not returned in our searches were also considered. Published data highlighting the potential burden of dry eye disease (DED) both prior to and following cataract surgery were reviewed as well as studies highlighting the effects of cataract surgery on the ocular surface, intra-operative measures to reduce deleterious effects on the ocular surface and current evidence on the management options of post-operative dry eye.

## Results

A total of 145 publications were identified as relevant to the subject matter of DED and cataract surgery for this narrative review. These selected original peer-reviewed articles included 33 review articles, 4 systematic reviews, 4 meta-analyses, 27 randomized controlled trials (RCTs), 33 cohort studies (30 prospective, 3 retrospective), 1 retrospective case-control study, 7 cross-sectional studies, 13 laboratory-based studies, 23 case series (16 prospective, 7 retrospective) and a national audit report. Of these publications, our search could only identify 58 that directly addressed dry eye in relation to cataract surgery. These included 1 meta-analysis, 2 review articles, 24 RCTs trials, 12 prospective cohort studies, 7 prospective case-control studies (including 2 laboratory-based studies), 1 retrospective case-control study, and 10 case series (4 prospective and 6 retrospective).

## Prevalence of cataract surgery associated DED

The prevalence of DED after cataract surgery is unclear. Ishrat et al.^7^ reported clinical signs of DED in 9% of patients 4 weeks after surgery, while Miyake and Yokoi^11^ documented such problems in 31% at the same time period. In a prospective study of 100 patients, Dasgupta and Gupta^12^ found that at 12 weeks post-surgery, 100% of patients showed abnormalities in tear break up time (TBUT), Schirmer I tests and DED symptomatology, while a prospective study by Choi et al.^13^ indicated that at 3 months 27% of patients experienced persistent DED symptoms based on the Ocular Surface Disease Index (OSDI) questionnaire (Allergan plc, Irvine, CA), and this was associated with reduced TBUT, increased corneal fluorescein staining and meibomian gland drop out.

With regards to duration of DED events after cataract surgery, in a prospective study of 86 patients Iglesias et al.^14^ reported that 32% experienced symptoms of DED up to 6 months post-surgery. However, in a prospective study of 50 patients by Kohli et al.^15^ and a retrospective study of 96 patients by Cetinkaya et al.,^16^ the signs and symptoms of DED appeared to return to pre-operative levels at 3 months.

The situation is further complicated as pre-existing DED in patients with cataracts is frequent,^17,^^18^ with one study in a prospective series of 120 patients presenting with cataracts, reporting that 80% had at least 1 abnormal test indicative of ocular surface disease (OSD) prior to surgery.^17^ These findings are in keeping with those of a multi-centre prospective study of 136 patients which revealed that 77% had positive corneal staining and 63% TBUTs of less than 5 seconds prior to cataract surgery.^18^

It is important to note that persistent post-surgical discomfort which occurs after many surgical events such as laser refractive surgery, dental implants and genitourinary procedures, can manifest and overlap with DED after cataract surgery.^19^ In a study of 119 patients, Sajnani et al.^19^ reported post-operative discomfort in 34% 6 months post-surgery, with greater prevalence in women and those with autoimmune disorders, non-ocular chronic pain syndromes and usage of antihistamine, anti-reflux, anti-insomnia, anxiolytic and anti-depressant medications. In addition, the manifest symptomatology of DED has been shown to depend on many factors, with Szakats et al.^20^ indicating that the reporting of DED symptoms after cataract surgery may be more dependent on patient satisfaction than on clinical measures of dry eye. It is important to note, however, that the any post-operative discomfort from cataract surgery can be related to other anterior segment pathology such as blepharitis, keratitis and uveitis.^21^–^23^

Such studies demonstrate that while the association between DED and cataract surgery is significant, it is multifactorial and complex.

## The background of DED

DED is a complex disease process and this is highlighted by the lack of a globally agreed definition, which may perhaps explain the variation in data relating to DED in the existing literature.^5^–^7^ DED is defined by the Tear Film and Ocular Surface Society Dry Eye Workshop II (TFOS DEWS II) as

a multifactorial disease of the ocular surface characterized by a loss of homeostasis of the tear film, and accompanied by ocular symptoms, in which tear film instability and hyperosmolarity, ocular surface inflammation and damage, and neurosensory abnormalities play etiological roles.^6^

The definition by Asia Dry Eye Society^24^ states that ‘dry eye is a multifactorial disease characterized by unstable tear film causing a variety of symptoms and/or visual impairment, potentially accompanied by ocular surface damage’, while the Korean Corneal Disease Study Group defines DED as a disease of the ocular surface that is associated with tear film abnormalities, where patients have at least one clinical sign and symptom for diagnosis.^25^ Although the overlap between the various definitions can be appreciated, this lack of consensus demonstrates the complex nature of DED.^26^

DED is common, with a prevalence of up to 75% in some populations when only clinical signs are taken into consideration.^6,^^27,^^28^ The multifaceted aetiology of DED can make it a challenging condition to manage effectively. Risk factors for DED include age; female gender; Sjogren Syndrome; contact lens wear; meibomian gland dysfunction (MGD); ethnicity; and other genetic factors.^6,^^22,^^27,^^28^

There are two main sub-categories of DED: evaporative dry eye (EDE) and aqueous deficient dry eye (ADDE), with a degree of overlap between the two.^6,^^27,^^28^ In EDE there is a rapid rate of tear film evaporation from the ocular surface, whereas in ADDE there is a reduced secretion of tears from the lacrimal gland.^6,^^27,^^28^ In both situations there is a net increase in tear film hyperosmolarity,^6^ resulting in a series of pro-inflammatory signalling processes which contribute to the disease process.^6,^^28^–^31^ Patients with DED can suffer from an array of symptoms including grittiness, foreign body sensation, photosensitivity, epiphora and visual disturbances^5,^^27,^^28,^^32^ which can have a significant impact on QOL.^33^–^35^ A large cohort questionnaire study by Miljanović et al.^33^ showed that participants with a diagnosis of DED reported problems with common activities of daily living such as reading, driving and work-related activities compared with participants without the diagnosis. A utility assessment study to quantify the impact of DED on QOL showed utility scores in DED to be comparable to patients with angina or who undergo regular dialysis.^36^ DED can also have a negative impact on mental well-being, with a systematic review and meta-analysis by Wan et al. showing that patients with DED have higher rates of anxiety and depression compared to controls.^36^ DED has an economic burden,^37^ with a decision tree analysis in the United States estimating the annual cost of treating a single DED patient to be $11,302.^38^

A thorough history and clinical examination are essential for diagnosis and management of DED.^6,^^27^ For clinical diagnostic testing, the TFOS DEWS II recommend a dry eye symptomatology questionnaire, as well as the measurement of non-invasive tear break up time (NIBUT), tear film osmolarity, ocular surface staining, tear meniscus height and assessment for any MGD.^6,^^27^ With regard to patient symptomatology, there are several validated questionnaires available including the OSDI, Standard Patient Evaluation of Eye Dryness (SPEED), McMonnies, Dry Eye Questionnaire–5 (DEQ-5) and the Symptom Assessment in Dry Eye (SANDE) questionnaires.^39^–^43^ There can, however, be a discordance between symptomatology and clinical signs in DED, adding a further caveat in both the diagnosis and effective management of the condition.^44,^^45^ A systematic literature review highlighted that there is limited consistency between patient-reported symptoms and clinical signs, with Bartlett et al.^44^ suggesting that this may be due to poor correlations between the various clinical DED tests.

DED is clearly a common condition with significant effects on the QOL of patients and associated economic costs.^6,^^28,^^32^–^38,^^46^ The effective treatment of DED both prior to and following cataract surgery is important in ensuring that patients receive optimal care.^6,^^47,^^48^

## The pathophysiology of cataract surgery associated DED

**Figure 1.**
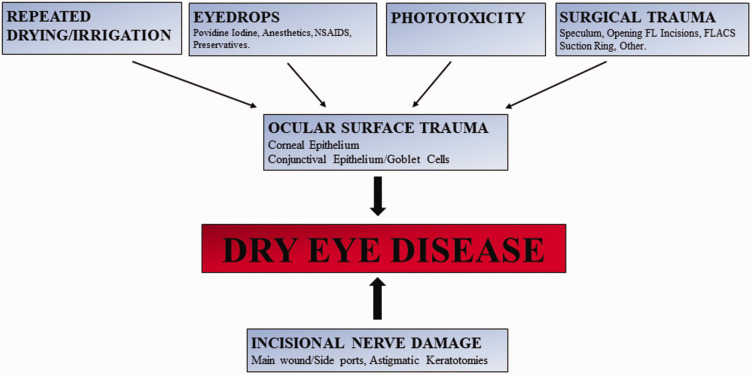
Pathophysiology of intra-operative factors in cataract surgery contributing to DED. FLACS: Femtosecond laser-assisted cataract surgery; FL: Femtosecond laser; NSAIDS: Non-steroidal anti-inflammatory drugs.

While the published evidence shows inconsistencies, probably due to the discordance between DED symptoms and signs,^44,^^45^ as well as the variability of current DED assessment methodologies, there clearly is an association between cataract surgery and both development of iatrogenic DED and exacerbation of pre-existing DED.^7–18,^^47^–^51^ As discussed, disruption in tear film homeostasis is a key component in the pathogenesis of DED and there are a number of possible intra- and post-operative factors in modern phacoemulsification cataract surgery (PCS) which can disturb the tear film milieu.^6^ These factors appear to include incisional corneal nerve injury and ocular surface damage from the toxic effects of components of eye drops; ocular surface drying/repeated irrigation; phototoxicity; and surgical trauma (Figure 1).

### Incisional corneal nerve damage

In laser kerato-refractive surgery the treatment of pre-existing DED, exclusion of patients with intractable DED and the routine use of post-operative lubricating drops are consistently undertaken to minimize DED due to corneal nerve damage induced during procedures such as laser in situ keratomileusis (LASIK) and photorefractive keratectomy (PRK),^52^–^54^ with patients being warned and consented pre-operatively of the occurrence of such problems. However, damage to radial corneal stromal nerves inflicted as a result of full-thickness incisional wounds in cataract surgery and subsequent disruption of tear film homeostasis^51^ do not appear to be fully appreciated.^55^

The incisional damage to corneal nerves in cataract surgery has been known for decades.^55^–^61^ Lyne examined 29 eyes undergoing ‘large’ incision cataract surgery and documented complete loss of sensitivity in the sector of cornea enclosed by the arc of the incision in all cases, with only 10% having normal sensitivity at 2 years.^56^ John,^57^ using a smaller scleral tunnel technique, measured corneal sensitivity in 60 eyes after PCS and reported decreased sensation over the incision width extending in a wedge-shaped sector over one month, which did not encompass the central cornea. Using a similar scleral tunnel technique, Kohlhass^58^ documented reduced sensitivity at 12 months extending to the centre of the cornea in 26 patients. Khanal et al.^59^ examined 18 patents who underwent PCS, with corneal incisions, albeit larger (3.0 mm extending to 4.1 mm) than those routinely used today (1.8 mm to 2.8 mm), and reported that nerve function had not returned to pre-operative levels at 3 months. Conversely, Kim et al.^60^ examined 40 patents who underwent PCS with 3.0 mm clear corneal incisions and found that nerve function appeared to return to near pre-operative levels at 3 months, although on confocal microscopy the basal sub-epithelial plexus was still reduced.

Further long-term investigations are indicated, but such studies denote that incisional wounds in cataract surgery, even with modern ‘small’ and so-called ‘micro-incisional’ techniques, where the incision widths are typically less than 2.0 mm, can disrupt radial stromal nerves at the limbus/corneal periphery, resulting in loss of sensation. This sensory loss may take months to return to normal and is likely to upset tear film homeostasis while compromised.^56^–^60^ As with laser kerato-refractive surgery, patients need to be warned of such potential complications in relation to cataract surgery and caution needs to be taken in those with severe pre-existing DED, corneal neurotrophic and lagophthalmic pathologies, with the addressing of such problems pre-operatively, limitation of incision size and careful post-operative management. It should be noted that in such situations it is not just full-thickness incisions that need to be considered but perhaps any partial thickness wounds to address astigmatism such as astigmatic keratotomies/limbal relaxing incisions due to the potential for corneal nerve damage/nerve transection,^61,^^62^ although further clinical research is indicated with regard to astigmatic incisions to more fully elucidate their relationship to DED after cataract surgery.

### Ocular surface damage due to the toxic effects of eye drops

The use of eye drops during and after surgery may lead to potentially harmful effects on the ocular surface with injury to corneal epithelial and conjunctival epithelial and goblet cells. Li et al.^63^ investigated 37 patients before and up to 3 months after surgery and documented DED after surgery in most, with the presence of conjunctival epithelial squamous metaplasia on impression cytology, especially in the region of the lower lid. They suggested that the peri-operative use of eye drops is one of the major pathogenic factors causing DED after cataract surgery.^63^

While the immediate pre-operative use of povidone-iodine has been shown to be important in reducing the risk of post-operative endophthalmitis,^64^ laboratory studies have demonstrated its potentially toxic effects on the corneal surface.^65,^^66^ Similar changes have been shown with the administration of topical anaesthetic drops.^67,^^68^ The type and volume of such drops should therefore be carefully considered, especially in eyes with pre-existing DED/OSD.

Following routine cataract surgery patients are typically given treatment regimens of topical steroid, non-steroidal anti-inflammatory and antibiotic drops as part of standard care. Such topical medications usually contain preservatives. Both laboratory and clinical studies have demonstrated the toxic properties of eye drop preservatives on the ocular surface.^69,^^70^ To investigate the effects of preservatives on DED after cataract surgery, Jee et al.^71^ randomized 80 patients to receive either post-operative preservative-free sodium hyaluronate 0.1% eye drops and preservative-free fluorometholone 0.1% eye drops, or eye drops containing preservatives. Two months after surgery, patients receiving preservative-free drops had better TBUTs, goblet cell counts, Schirmer I test, corneal fluorescein staining and OSDI questionnaire scores.^71^ Such results support the use of preservative-free drops after cataract surgery to minimize DED, at the very least in those with pre-existing ocular surface pathology/DED.

### Repeated intra-operative ocular surface drying and irrigation

During cataract surgery, the ocular surface is exposed and at risk of cycles of repeated drying followed by irrigation to maintain surgical optical clarity. As such there is potential for damage to the corneal and conjunctival surface. He et al.,^72^ in a prospective randomized controlled study (RCT) of 149 patients, compared the intra-operative use of hydroxypropyl methylcellulose (HPMC) 2% or balanced salt solution (BSS) to coat the ocular surface during cataract surgery and documented that when using HPMC 2% intra-operatively, tear film assessment measures (Schirmer I test) and ocular surface health (fluorescein staining) were better in some patient groups, especially those with DED before surgery and patients whose surgical time was protracted.^72^ Similarly, Yusufu et al.,^73^ in a prospective interventional case series of 60 eyes, documented reduced DED symptoms and better TBUT with the intra-operative use of HPMC 2% compared to BSS. Likewise, Yoon et al.^74^ in a comparative prospective study of 24 patients, reported the use of an ophthalmic visco-surgical device (OVD) (DisCoVisc, Alcon Laboratories, Inc.) intra-operatively to coat the ocular surface and found significant improvements in TBUT, OSDI scores and ocular surface staining scores at 1 week post-surgery. These improved outcomes with ocular surface coating with OVDs are supported by the studies of Oh et al.^51^ and Moon et al.^75^ Oh et al.^51^ documented a correlation between cataract surgery operative time and mean goblet cell density cell loss at 1 day and 1 and 3 months post-operatively, suggesting that prolonged intra-operative exposure is important in resultant ocular surface damage. Moon et al.^75^ undertook a prospective case series of 58 patients and showed that compared to a non-aspirating speculum, the use of an aspirating speculum, which removes fluid from the ocular surface during cataract surgery, led to reduced TBUT and higher OSDI scores at 1 week after surgery.

Such investigations intimate that intra-operative ocular surface drying and need for frequent irrigation during cataract surgery are important factors in the pathophysiology of cataract surgery-related DED, and that the use of OVDs to coat the ocular surface intra-operatively and limitation of operative times are important to limit ocular surface damage, especially in patients with pre-existing DED.

### Phototoxicity

The phototoxic effects of the operating microscope light on the ocular surface have been demonstrated in both laboratory and clinical studies.^76^–^78^ An in-vivo study examining the effects of microscopic light on rabbit eyes demonstrated damage to corneal and conjunctival epithelial cells, reduced aqueous tear production and a reduction in goblet cell density,^76^ while an in-vitro study on porcine conjunctival cells showed that microscope light exposure resulted in fibroblast cell damage and delayed wound healing.^77^ In a prospective clinical study by Cho et al, duration of microscopic light exposure during cataract surgery was associated with worse DED symptoms, TBUT, Schirmer I test and tear meniscus height in patients with no prior history of dry eyes.^78^

To what degree phototoxicity and the factors described above (e.g. intra-operative ocular surface drying and need for frequent irrigation) contribute separately or collectively to the pathogenesis of cataract surgery associated DED is uncertain, and warrants further study, with investigations aimed at analysing these factors individually. However, in cases at risk of DED after cataract surgery it is sensible to limit intra-operative light exposure as much as possible to prevent damage to the ocular surface and possible subsequent DED-related problems.

### Femtosecond laser-assisted cataract surgery (FLACS) and DED

It has been a decade since the introduction of FLACS.^79^ While it offers potential advantages in terms of automation of some of the surgical steps of cataract surgery, clinical outcomes appear to be no better than those with PCS.^80,^^81^ At present there is limited evidence on the long-term effects of FLACS on the ocular surface, although there are a number of studies which indicate that akin to PCS, FLACS also leads to signs and symptoms of DED.^82,^^83^

In a prospective case series of 38 patients undergoing FLACS, Ju et al.^82^ reported that TBUT and Schirmer I test were both reduced at 1 week but returned to pre-operative levels at 1 and 3 months, respectively; corneal staining and OSDI symptoms scores increased after surgery, however, and did not return to baseline levels at 3 months, with the authors speculating that this may be due to damage to corneal limbal stem cells and conjunctival goblet cells by the FLACS suction ring. In a non-randomized comparative cohort study^83^ of 137 patients undergoing FLACS or PCS, Yu et al. documented worsening of corneal fluorescein staining, Schirmer I test, TBUT and OSDI scores at 1 month, with corneal staining and OSDI scores significantly worse in the FLACS treated group. Similarly, a RCT by Shao et al.^84^ in 300 eyes (150 eyes undergoing FLACS, 150 eyes undergoing PCS) demonstrated worse corneal staining and OSDI scores with FLACS at 1 week, although these changes had returned to pre-operative levels by 3 months.

It appears from these limited studies that FLACS may have a slightly greater propensity to cause DED compared to PCS, This may be attributable to additional ocular surface trauma from the FLACS suction ring damaging limbal corneal/conjunctival epithelial cells and conjunctival goblet cells, epithelial/stromal trauma from the use of a Sinskey hook to open the femtosecond laser corneal incisions, extended surgical times required for the laser application, administration of pre-operative topical non-steroidal anti-inflammatory drops to maintain pupillary dilatation or an as yet unidentified factor. These factors require further investigation but imply that caution needs to be taken with FLACS in patients with severe pre-existing DED.

### Special considerations: Multifocal intraocular lenses (MIOLs)

Advances in small-incision PCS have been accompanied by increasing patient and surgeon expectations.^4,^^5^ This is especially true with the use of MIOLs^85^ where patients often undergo the procedure without symptomatic cataract in order to reduce dependence on spectacles, paying a premium for such lenses. As the first refractive component of the eye, an intact, healthy pre-corneal tear film is prerequisite for optimal visual performance.^10^ This is especially imperative in patients receiving MIOLs, where the compromises inherent in the optics of such lenses can be amplified by irregularities in other refractive components of the eye.^86^–^88^ Studies have shown DED to be a significant contributory factor in patient dissatisfaction following surgery.^86^–^88^ In a retrospective review by Woodward et al.^86^ in 32 patients with MIOL implantation, DED was considered to be the cause of impaired vision in 15% of cases and the cause of photic phenomena in 2%. Similarly, reduced visual performance was attributed to DED in 35% of cases in a retrospective review of 49 patients by Schallhorn et al.,^87^ while a double-masked RCT by Donnenfeld et al.^88^ reported that eyes which received cyclosporine 0.05% drops to optimize tear film function after MIOL implantation had better TBUT, contrast sensitivity and conjunctival staining assessments compared to those that only administered lubricating eye drops. Because of such considerations, it is imperative that pre-existing DED is diagnosed and corrected prior to cataract/refractive lens exchange surgery with MIOL implantation to optimize visual outcomes and patient satisfaction.^86^–^88^ Indeed, akin to kerato-refractive laser surgery, given the optical considerations and high patient expectations, patients with intractable DED are not suitable for MIOL implantation.

## Considerations of the management of cataract surgery associated DED

### Pre-operative management of DED

#### Background

The pre-operative diagnosis and management of pre-existing DED are important prior to cataract surgery due to the potential for inaccurate biometric and corneal topographic assessments due to tear film irregularities^7,^^11–18^ as well as exacerbation of the problem after surgery ^7,^^11–18,^^47,^^48^ (Table 1). Given the typical age groups, patients presenting for cataract surgery often have pre-existing DED, with Gupta et al.,^17^ in a prospective case series of 120 such patients, reporting that 80% had at least one abnormal tear film measurement parameter. The exacerbation of pre-existing DED has been demonstrated in murine models of DED, with increased corneal lymphgiogenesis, neovascularization and inflammation after cataract surgery compared to non-dry eye models.^89^ Aggressive pre-operative DED management in high-risk cases with severe pre-existing DED may limit further exacerbations. In a retrospective review of 72 eyes in 41 patients with a background of graft versus host disease, Franco et al.^90^ documented that following careful pre-operative management of DED, overall signs and symptoms did not change significantly after cataract surgery, although despite aggressive treatment of DED before and after surgery there were still two cases of corneal ulceration and perforation in these eyes with severely compromised ocular surfaces.

**Table 1.** Steps to reduce the risk of cataract surgery-related DED, based on current published literature.

Pre-operative	Intra-operative	Post-operative
Assess for DEDAssess for OSD• Treat pre-existing• DEDMGD	Limit incisional damage• ‘Micro’-incisional surgery• Consider avoiding AKs with DED/OSDLimit drop exposure• Avoid XS topical anaesthetic application• Single pre-op. drop of Povidine 5%• Care with pre-op NSAIDS in those with DED• PF drops in those with DED/OSDLimit repeated drying/irrigation• Consider coating OS with dispersive OVD ○ All those with DED ○ As a routine• Limit surgical time where possibleLimit Phototoxicity• Reduce surgical time/exposure• Microscope illumination ○ Adequate not excessiveLimit surgical trauma• Careful insertion of speculum• Care with FLACS suction ring• Avoid epithelial trauma• Opening FLACS incisions• Limit holding eye with forceps	Assess for DED• Avoid XS drop regimens• Consider PF drops• Lubricating drops/ointment• Management of MGD ○ Lid hygiene ○ Tea tree oil ○ Omega III ○ Topical Azithromicin ○ Systemic tetracycline• Topical Cyclosporin• Other anti-inflammatories ○ Lifitegrast• Mucin Secretagogues• Care with NSAIDs ○ Avoid with DED• Punctal Plugs

DED: dry eye disease; MGD: Meibomian Gland Dysfunction; OSD: Ocular surface disease; NSAIDs: Non-steroidal Anti-inflammatory drugs; FLACS: Femtosecond laser-assisted cataract surgery; OVD: Ophthalmic Viscosurgical Device; XS: Excess; PF: Preservative Free; OS: Ocular Surface.

The diagnosis and effective management of DED can be challenging^6,^^44^ due to the discordance between DED symptoms and clinical signs, and limitation of current methodologies of DED assessment. Sixty-eight ophthalmic practitioners who were surveyed on their single preferred choice of test for tear film examination indicated patient history and/or use of a dry eye questionnaire to be the first choice in 28% of responses, with the next three top choices being TBUT (19%), fluorescein staining (13%) and Rose Bengal staining (10%).^91^ In a retrospective case review of 467 patient notes where a new diagnosis of DED was made, the most frequent two-test combination was patient symptoms and corneal fluorescein staining in 44% of cases.^92^ While there is a plethora of clinical tests to assess for possible DED,^6^ most have limited diagnostic ability and repeatability.^93,^^94^ In a study of 75 DED patients by Nichols et al.,^93^ although patient-reported symptoms were moderately repeatable over two separate clinic visits, clinical tests such as corneal staining and assessment of the meibomian glands had limited repeatability. Sullivan et al.,^94^ in a longitudinal observational case series, documented that over 3 months of tear film osmolarity had the least variability compared to other clinical assessments such as Schirmer I test, TBUT and OSDI symptom scores.

A recent questionnaire analysis on dry eyes in cataract and refractive surgery conducted by the American Society of Cataract and Refractive Surgery (ASCRS) indicated that despite clinicians acknowledging the importance of DED management prior to cataract surgery, there was wide variety in treatment practices and clinical opinions.^55^ The ASCRS committee therefore devised a pre-operative screening algorithm, including a new pre-operative screening questionnaire (SPEED II), based on the Johnson & Johnson Vision Inc. SPEED questionnaire, and technician-performed, objective, non-invasive, point-of-care clinical diagnostic tests of tear osmolarity and tear film matrix metalloproteinase 9 (MMP-9) levels.^55^ This algorithm is commendable in that it is simple and can be easily incorporated into practice workflow both in the private and public health sectors and has the potential to identify DED prior to surgery, allowing its appropriate management and adequate patient counselling of potential DED problems, hopefully improving post-operative outcomes and patient satisfaction.

#### MGD and cataract surgery

MGD is common and can contribute to DED^6,^^95^–^98^ (Table 1). A prospective case series by Han et al.^99^ showed that following cataract surgery there are increased lid margin abnormalities at 3 months. The assessment and pre-operative management of any MGD are therefore important prior to cataract surgery. Treatment regimens for MGD include the regular use of warm compressors, lid hygiene, treatment of demodex and the administration of systemic tetracycline antibiotics and topical azithromycin.^6,^^95^–^97^ Such pre-operative interventions are supported by a recent RCT by Song et al.,^97^ in which 120 patients with moderate MGD, undergoing cataract surgery, were randomized into three cohorts. Cohort 1 received routine post-operative anti-inflammatory treatment (tobramycin/dexamethasone drops four times daily for one week, which was tapered over the following four weeks), Cohort 2 was prescribed pre-operative treatment of MGD including lid hygiene, warm compressors and the routine post-operative anti-inflammatory regimen as for Cohort 1, while Cohort 3 received a more intense and extended post-operative anti-inflammatory regimen (tobromycin/dexamethasone drops 6 times daily for 1 week and tapering over the following 4 weeks). Following surgery, the best outcomes in terms of MGD and DED scores both at 1 and 3 months were seen in Cohort 2, highlighting the importance of pre-operative treatment of MGD associated DED97 before cataract surgery.

#### Lubricating eye drops

The use of lubricating eye drops (including gels or ointments at night) and punctal plugs are effective in treatment of both ADDE and EDE^6,^^27,^^28^ Table 1. There is scarce evidence in the published scientific literature on the sole effects of the implementation of pre-operative lubricating drops on DED symptoms and signs after cataract surgery. In a prospective study by Ganesh et al.^100^ investigating the effects of a topical cyclosporine drops on DED after cataract surgery, one of the study arms consisted of patients receiving lubricating eye drops four times daily for 2 weeks prior to surgery, followed by a further course for 3 months after surgery. Compared to the control group, which received no lubricating drops, this group did not show worsening OSDI scores at three months, and tear film parameters returned to pre-operative levels despite worsening in the early post-operative period, while the control group’s (no lubricants) OSDI scores and abnormal tear parameters were still worse at 3 months.^100^ Clearly the importance of the pre-operative administration of ocular lubricants in reducing DED after cataract surgery requires further investigation.

### Intra-operative management of DED

As discussed above, there are several intra-operative factors that may be important in the pathophysiology of cataract surgery-induced iatrogenic DED including corneal nerve damage secondary to surgical incisions; the potentially toxic effects of povidone-iodine and topical anaesthetic drops on the ocular surface; repeated ocular surface drying/irrigation; phototoxicity; and direct ocular surface trauma.^51,^^55–78,^^83–85^ The cataract surgeon needs to be mindful of these factors when operating on patients, especially when a patient has any pre-existing DED ^17^,^18,^^47^,^48,^^97^(Table 1).

Incision sizes in modern PCS cataract surgery are typically less than 3.00 mm, but micro-incisional techniques (under 2.00 mm) may cause less disruption in their neurotrophic and DED potentiating effects and requires further investigation. Astigmatic keratotomy incisions, both penetrating and partial thickness, require careful surgical planning both with respect to refractive outcome and to pre-existing DED problems. In cases with severe DED, surgeon’s use of the larger incision astigmatic keratotomies/LRIs should be carefully considered in order to limit additional corneal nerve trauma,^61,^^62^ and alternatives such as toric intraocular lenses should be considered. The use of a dispersive OVD such as HMPC 2% on the ocular surface during cataract surgery may have protective effects, with prospective randomized and non-randomized studies showing benefits in reduced symptoms and signs of DED post-operatively^72^–^74^ and it is the authors’ choice to coat the ocular surface with an OVD in eyes with pre-existing DED during cataract surgery. Finally, to limit post-operative DED it is sensible that the use of any peri-operative topical medications needs to be both appropriate and not excessive, direct ocular surface trauma is kept to a minimum with careful tissue handling, operative light exposure is appropriate for surgical visualization and not excessive, and operative/ocular exposure times are minimized by careful surgical planning. The latter is important in the selection of cases for teaching purposes where operative times are often extended.

### Post-operative management of DED

#### Optimization of standard treatment regimens

As part of standard care following cataract surgery, patients are given courses of topical steroid, non-steroidal anti-inflammatory and antibiotic drops for about 1 month. The use of preservative-free eye drops regimens has been shown to be beneficial with one RCT reporting improved post-operative TBUTs, goblet cell counts, Schirmer I test, fluorescein staining and OSDI scores with preservative-free drops.^63^ While such preservative-free regimens are associated with increased expense, as well as some patient inconvenience in terms of individual vial drop administration, they are a useful adjunct and the authors’ preferred choice for patients undergoing cataract surgery with pre-existing DED (Table 1). Further research is required to support their usage and perhaps eventual universal introduction.

In their RCT, Song et al.^97^ investigated a treatment arm in which some patients received a more intense and extended post-operative anti-inflammatory regimen (tobromycin/dexamethasone drops 6 times daily for 1 week and tapering over the following 4 weeks). In this cohort, TBUTs, ocular symptom scores and lid margin, and meibum quality were better than those receiving the standard post-operative anti-inflammatory regimen.^91^ Such results suggest that more intensive corticosteroid regimens, with careful intraocular pressure monitoring, may be beneficial for those patients with pre-existing DED or who develop DED after cataract surgery and merit further investigation.

#### Lubricating eye drops

There have been several studies looking at management of post-operative DED with lubricant eye drops following cataract surgery.^101–104^ In a RCT of 48 patients undergoing cataract surgery by Sánchez et al.,^101^ patients received either standard care treatment of tobramycin/dexamethasone drops post-surgery or standard care treatment plus additional preservative-free Systane lubricating drops (Alcon Laboratories Inc) four times daily for a month. At 1-month follow-up patients in the intervention arm had superior TBUTs, OSDI symptom scores, and lower inflammatory markers on their conjunctival cytology samples.^101^ Similarly, an observational study of 36 patients by Stefan and Dumitrica revealed that those receiving post-operative Systane drops had better Schirmer I test, TBUTs and subjective symptom scores compared to controls.^102^ In a prospective study comparing the effect of two different lubricating drops after cataract surgery (carbomer sodium hyaluronate trehalose (CHT) eye drops and sodium hyaluronate eye drops), Valerio et al.^103^ reported that the CHT group had better TBUT and OSDI scores, as well as greater patient satisfaction following surgery at 3 weeks. Handayani et al.^104^ in a RCT of 38 patients investigated the consequences of post-operative vitamin A eye drops on goblet cell density following PCS compared to a placebo (lubricating drops not containing vitamin A). At 4 week review impression cytology showed the mean goblet cell density to be higher in the group receiving vitamin A, suggesting a protective effect for vitamin A against goblet cell loss after cataract surgery.^105^

While these studies have small sample sizes, they support the positive effects of post-operative lubricating drops in reducing DED symptoms and signs after cataract surgery. It is clear, however, that significant additional research is required to optimize post-operative lubricant drop regimens with regards to both their preferred components and the duration/frequency of the administration regimen.

#### Topical cyclosporine drops

Inflammation is a key component in the pathophysiology of DED.^6,^^27^,^28,^^30^,^31,^^48^ Cyclosporine, a peptide originating from fungi, has immunosuppressive effects by inhibiting T-Cell activation and subsequent downstream pro-inflammatory sequelae.^105–107^ Cyclosporine eye drops have been shown to be effective in the management of DED.^105–108^ Findings from a prospective study suggested that topical cyclosporine may have protective effects on the ocular surface after cataract surgery.^100^ Ganesh et al.^100^ randomized 67 patients to three treatment regimen arms: pre-operative lubricating drops 4 times daily and 0.05% topical cyclosporine twice daily for 2 weeks followed by a further 3-month course post-operatively; pre-operative lubricating drops 4 times daily for 2 weeks followed by a further 3 month course post-operatively and a standard care control group with no lubricant/cyclosporin drops. All three groups received the standard post-operative treatment of corticosteroid, Nepafenac and Moxifloxacin eyedrops. At 3 months patients receiving the combination of cyclosporine and lubricating drops had better scores on OSDI, TBUTs, Schirmer I test, and tear osmolarity compared to pre-operative levels. In another RCT of 30 patients by Hamada et al.,^107^ those receiving cyclosporine drops 0.05% rather than carboxymethylcellulose 0.5% had better tear osmolarity, TBUTs, Schirmer I test and enhanced corneal nerve recovery at 1 month after cataract surgery. Similarly, the beneficial tear film effects of post-operative cyclosporine drop 0.05% was seen in prospective study of 32 patients by Chung et al.^108^ Such studies show promising results and the addition of cyclosporin drops appears to be beneficial and may be a useful adjunct in those patients with pre-existing DED. Further studies are required to optimize treatment regimens and to investigate whether pre-operative administration of cyclosporin drops might be beneficial.

#### Mucin secretagogues

Loss of conjunctival goblet cells contributes to the development of DED6 and cataract surgery has been shown to cause goblet cell drop out.^6,^^51^ Goblet cells secrete mucins which have roles in maintaining ocular surface lubrication and in the removal of debris from the ocular surface.^109^,^110^ The two mucin secretagogues eye drops, diquafosal and rebamipide, are now approved for use in Japan and South Korea for the treatment of DED and studies have recently demonstrated their efficacy in the treatment of DED following cataract surgery.^111^

Diquafosal is a P2Y2 receptor agonist and laboratory studies have shown that it can increase mucin secretion and reduce the rate of corneal epithelial cell loss.^112^,^113^ In a RCT by Miyake and Yokoi in 154 eyes,^11^ patients with DED following cataract surgery were randomized to receive either Diquafosal 3% drops 6 times a day for 4 weeks or artificial tears 6 times a day for 4 weeks. At 4 weeks the TBUT was longer in the Diquafosal group, although subjective symptom scores were better in the artificial tears group. Cui et al.^114^ randomized 94 eyes with pre-existing DED to receive either Diquafosal 3% or 0.1% sodium hyaluronate drops after cataract surgery. At 12 weeks TBUTs, goblet cell density, and OSDI symptom scores were all superior in the Diquafosal group. Similar findings showing the superiority of Diquafosal in the management of post-operative DED compared to artificial tears have been documented in other RCTs.^115–117^ The results of a recent meta-analysis of these RCTS by Zhao et al.^118^ suggested that topical diquafosal 3% is more effective than artificial tears in the post-operative management of DED. At present this medication is not available in Europe but given its actions in reducing conjunctival goblet cell loss, which is known to occur after cataract surgery,^6,^^51^ hopefully its introduction will help in the management of cataract surgery associated DED.

Rebamipide is a derivative of quinolone antibiotics and was initially approved for the treatment of gastric ulcer disease.^119^ Laboratory-based studies have shown rebamipide to have protective effects on the preservation of the corneal epithelium as well as in improving tear film stability.^120^,^121^ An in vivo study has suggested that it may protect the corneal epithelium from povidone-iodine toxicity.^122^ Several clinical studies have shown rebamipide to be effective in improving tear film stability in DED.^122–125^ In a recent RCT, rebamipide was found to have a protective effect against conjunctival goblet cell loss in patients receiving concurrent diclofenac.^126^ As yet there are no studies investigating its protective effects against cataract associated DED but given its action it may well be beneficial and investigative studies are indicated in this respect.

#### Other treatment interventions

##### Anti-inflammatory agents: Lifitegrast

Lifitegrast is an integrin (lymphocyte function-associated antigen 1 (LFA-1) antagonist preventing it from binding to the intercellular adhesion molecule 1 (ICAM-1); this causes down regulation of inflammation mediated by T-lymphocytes.^127^ It has a rapid onset of action, possibly as a result of multi-target action on the inflammatory cycle, and it has ability to modulate already active T-cells.^128–130^ It has been shown in multiple clinical trials to be effective for the treatment of DED^128–130^ including severe cases such as those with graft versus host disease.^131^ In such studies it has been shown to significantly improve inferior corneal fluorescein staining scores and eye dryness scores, with limited adverse effects such as ocular irritation and dysgeusia.^128–130^ There are as yet no published studies of its usage after cataract surgery in the prevention of DED, but Hovanesian et al.^132^ have reported improvements in higher-order aberrations, accuracy of pre-operative biometry, as well DED symptom scores, TBUTs and corneal fluorescein staining in patients using lifitegrast. Further clinical investigations are indicated to investigate its role in the management of cataract surgery associated DED.

##### Omega-3 supplementation

There is evidence to suggest that omega-3 fatty acid supplementation may improve the signs and symptoms of DED, with a recent meta-analysis of randomized controlled trials by Giannaccare et al. indicating that omega-3 supplementation may be effective in the management of DED.^133^ In the context of DED post-cataract surgery, a RCT by Mohammadpour et al.^134^ indicated that compared to controls receiving conventional post-operative treatment, patients receiving omega-3 supplements (1000 mg 8 hourly for 1 month) after cataract surgery had better OSDI scores and higher TBUTs, suggesting that this may be a useful adjunctive therapy for those patients with newly diagnosed DED after cataract surgery as well as for those with pre-existing DED undergoing surgery.

##### Lactoferrin

Lactoferrin is a glycoprotein found in the tear film that has both anti-inflammatory and antimicrobial actions.^135^,^136^ There is some evidence to suggest that lactoferrin supplementation may have a role in the management of DED.^137^,^138^ In a RCT on 58 eyes undergoing cataract surgery, Devendra et al.^139^ reported that patients receiving oral lactoferrin supplements were found to have lower OSDI scores, and higher TBUT and Schirmer I tests compared to controls.

##### Tea tree oil lid scrubs

Tea tree oil has antimicrobial properties and has been used in the treatment of MGD, where there can be a buildup of demodex on the eyelids.^140–142^ A RCT by Mohammadpour et al.^143^ showed that compared to controls, scrubbing the eyelids with a shampoo containing tree oil after phacoemulsifiaction cataract surgery led to better OSDI scores, tear osmolarity and TBUT scores, as well as lower counts of eyelid demodex.

##### Bandage contact lenses

Bandage contact lenses are commonly used in ophthalmology for the management of persistent epithelial defects^144^ and are generally not used following cataract surgery due to the risk of post-operative microbial keratitis. In an RCT on 40 patients undergoing cataract surgery, Shi et al.^145^ randomized patients to receive a bandage contact lens for 1 week post-surgery, with the control group just wearing an eye pad for 1 day post-surgery. Post-operative drops regimens were the same for both groups. At 1 week, review patients in the bandage contact lens group had higher TBUTs and tear meniscus height with no post-operative complications.^145^ Further studies are necessary to determine whether the routine use of bandage lenses, with antibiotic cover, in the early post-operative period might be of benefit after cataract surgery.

## Conclusion

DED is a common condition and can be induced and aggravated by cataract surgery, with a considerable consequence on the QOL of patients.^6^,^7,^^33–35^ A significant number of patients may present to the cataract pre-assessment clinic with pre-existing DED and due to the discordance between clinical signs and patient symptoms, clinicians are faced with challenges in the effective diagnosis and management of this condition.^16–18,^^44^,^45^ Pre-operative assessment for pre-existing DED is essential to ensure that patients receive appropriate pre-operative DED treatment prior to cataract surgery and to ensure that accurate measurements are obtained in terms of biometry and corneal topography/tomography for surgical planning. Cataract surgeons should be mindful of the detrimental intra-operative effects of cataract surgery on the ocular surface and take steps to limit these. Post-operative management of DED is crucial in ensuring that tear film homeostasis is preserved as much as possible and to avoid long-term adverse effects of the ocular surface. Considering the great regularity with which cataract surgery is undertaken,^1–3^ the associated high patient expectations^4^,^5^ and high frequency of occurrence of DED^21^,^22^ the finding of only 58 publications directly addressing DED in relation to cataract surgery is surprising and suggests that this problem might be overlooked by clinicians and requires much further investigation.

## Financial/proprietary interests

Professor O’Brart holds non-commercial research grants from Alcon Laboratories, Inc., Rayner Ltd and Avedro Inc.
